# A fast and reliable method for monitoring genomic instability in the model organism *Caenorhabditis elegans*

**DOI:** 10.1007/s00204-021-03144-7

**Published:** 2021-08-30

**Authors:** Merle Marie Nicolai, Barbara Witt, Andrea Hartwig, Tanja Schwerdtle, Julia Bornhorst

**Affiliations:** 1 Present Address: Food Chemistry, Faculty of Mathematics and Natural Sciences, University of Wuppertal, 42119 Wuppertal, NRW Germany; 2 Present Address: Department of Food Chemistry, Institute of Nutritional Science, University of Potsdam, Nuthetal, 14558 Brandenburg, Germany; 3 Present Address: Department of Food Chemistry and Toxicology, Institute of Applied Biosciences, Karlsruhe Institute of Technology (KIT), 76131 Karlsruhe, Baden-Württemberg Germany; 4 TraceAge-DFG Research Unit on Interactions of Essential Trace Elements in Healthy and Diseased Elderly (FOR 2558), Berlin-Potsdam-Jena-Wuppertal, Germany

**Keywords:** Alkaline unwinding, Genomic instability, *Caenorhabditis elegans*

## Abstract

The identification of genotoxic agents and their potential for genotoxic alterations in an organism is crucial for risk assessment and approval procedures of the chemical and pharmaceutical industry. Classically, testing strategies for DNA or chromosomal damage focus on in vitro and in vivo (mainly rodent) investigations. In cell culture systems, the alkaline unwinding (AU) assay is one of the well-established methods for detecting the percentage of double-stranded DNA (dsDNA). By establishing a reliable lysis protocol, and further optimization of the AU assay for the model organism *Caenorhabditis elegans (C. elegans),* we provided a new tool for genotoxicity testing in the niche between in vitro and rodent experiments. The method is intended to complement existing testing strategies by a multicellular organism, which allows higher predictability of genotoxic potential compared to in vitro cell line or bacterial investigations, before utilizing in vivo (rodent) investigations. This also allows working within the 3R concept (reduction, refinement, and replacement of animal experiments), by reducing and possibly replacing animal testing. Validation with known genotoxic agents (bleomycin (BLM) and *tert*-butyl hydroperoxide (tBOOH)) proved the method to be meaningful, reproducible, and feasible for high-throughput genotoxicity testing, and especially preliminary screening.

## Introduction

Maintenance of genome integrity is an organism’s top priority to ensure a healthy life and successful reproduction of the species (Jackson and Bartek 2009). The genomic DNA is under constant attack of extrinsic and intrinsic genotoxic agents which may result in genetic alterations in somatic and/ or germ cells, which in turn might manifest target place-dependent negative outcomes, such as impaired transcription or replication, apoptosis, or necrosis, or fixation to mutation. This potentially will lead to cancer and non-cancer genetic diseases for somatic cells and infertility, heritable damage and intergenerational genetic diseases for germ cells (Erickson 2010; Kawanishi et al. 2006; Lindahl and Barnes 2000; Valko et al. 2006). The purpose of genotoxicity testing is to identify such genotoxic agents and their potential for genotoxic alterations. The testing is part of approval procedures and testing strategies of the chemical and pharmaceutical industry, as well as risk assessment of food ingredients. Due to the wide potential spectrum of DNA damages, it is imperative to consider multiple endpoints to systematically assess genotoxicity. As defined in the OECD guidelines, genotoxicity testing includes assays that measure direct, irreversible damage to the DNA that is transmissible to the next generation (i. e. mutagenicity), as well as tests that evaluate directly the induced DNA damage that may or may not result in permanent alterations (and is therefore no direct evidence of mutagenicity) (OECD 2016). In both cases, in vitro and in vivo models (mostly rodents) are used and guidelines recommend a test battery starting with testing for gene mutation in bacteria, followed by in vitro assays using mammalian cell lines, before recommending an in vivo test system (Committee 2011; EMA/CHMP/ICH 2011; Phillips and Arlt 2009). Although these tests are routinely used, they present crucial limitations (i. e. lack of xenobiotic metabolism and bacteria-specific reactions (Yasui et al. 2021), use of tumor cells), which affect the usefulness of the assays to predict the genotoxic potential of a substance in vivo. With the emergence of a stronger awareness of animal welfare in scientific experiments, classic and well-established in vivo studies are increasingly attempted to be replaced by equally meaningful tests, which follow the 3R (refine, reduce, replace) principle (Doke and Dhawale 2015; Freires et al. 2017). For this purpose, as well as to overcome present limitations, current efforts include the usage of 3-D cell culture models or alternative in vivo model organisms. Within this study, the model organism *C. elegans* was applied for genotoxicity testing. The well-established in vitro method of DNA alkaline unwinding (Doke and Dhawale 2015; Elsakrmy et al. 2020) was optimized for detecting the proportion of dsDNA in *C. elegans* and was verified using validated genotoxins to provide a rapid genotoxicity evaluation in a metazoan organism.

## Materials and methods

### Worm cultivation and exposure to positive controls

For assay development, the wild type (WT) N2 Bristol *C. elegans* strain was used, which was provided by the *Caenorhabditis Genetic Center* (CGC; University of Minnesota).

Worms were cultivated on agar plates at 20 °C as described by Brenner (1974). After synchronization and hatching, L1 (larval stage 1) worms were seeded on OP50 *E. coli* covered NGM plates until the population reached L4 without further interference. For treatment, tBOOH and BLM were used as positive controls in ranges of 0–5 mM tBOOH and 0–80 µM BLM for 1 h. BLM sulfate was purchased from Selleckem (NSC125066) and tBOOH from Merck (CAS 75-91-2). Both chemicals were used as received and diluted in 85 mM NaCl to the desired concentration. 3000 L4 larvae were incubated in liquid (85 mM NaCl) in the absence of *E. coli* while rotating slowly to ensure equal substance uptake. Afterward, samples were washed at least three times with 85 mM NaCl + 0.01% Tween before continuing with the survival or alkaline unwinding assay.

### Survival

The toxicity of the substances was determined using the survival assay as described previously (Helmer et al. 2021). After treatment, a specific number of worms were transferred to OP50-seeded NGM plates. Alive and dead worms were manually counted 24 h post-treatment. The vitality of the animals was checked via the mechanical stimulus of touch using a platinum/zirconium wire, which stimulates the worms to move. Any worms that did not respond to the stimulus were considered dead.

### Adapted alkaline unwinding for use in *C. elegans*

After treatment, worms were placed in 1 mL alkaline unwinding buffer (AU buffer; 0.5 M NaH_2_PO_4_, 0.5 M Na_2_HPO_4_, 0.1 M EDTA, pH 7.5). To reduce possible additional strand breaks during sample preparation, samples were kept on ice at all times and all experimental steps were performed in the dark. Worms were made assailable for the alkaline solution using slight sonification and a large liquid volume (UP100H ultrasonic processor (Hielscher), 1 mL AU buffer, 2 × 20 s on lowest setting, 100% amplitude). After centrifugation (1400 rpm, 2 min, 4 °C) and removal of the supernatant, 1.5 mL alkaline solution (0.9 M NaCl, 10 mM Na_2_HPO_4_, 0.03 N NaOH in dH_2_O) was added to all samples. The DNA was allowed to unwind at RT in the dark for exactly 15 min, before neutralizing the solution with 0.1 N HCl (exact volume was adapted to reach pH 6.8 ± 0.02), sonification on ice (15 s, highest setting), and adding SDS to a final concentration of 0.05%. The single- and double-stranded DNA were separated by successional elution of 0.15 M and 0.35 M potassium phosphate buffer over 1 mL hydroxyapatite columns at 60 °C. The amounts of DNA for the single-stranded DNA (ssDNA) and dsDNA fraction were determined using Hoechst stain (Hoechst 33258 nucleic acid stain) at a final concentration of 7.5 × 10^–7^ M and the fluorescence was measured using a microtiter fluorescence reader (Infinite Pro, Tecan, Switzerland; 360 nm excitation wavelength and 455 nm emission wavelength). As described by Hartwig et al. 1993, staining availability of Hoechst was tested, which allowed calculations for dsDNA fraction (Hartwig et al. 1993). For this, the fluorescent signals of ssDNA and dsDNA were compared, which yield 0.4 × lower values for dsDNA compared to ssDNA, resulting in the following calculation (formula 1). Statistical evaluation was performed using Graph Pad Prism 9.

1 $${\text{dsDNA}} = \frac{{\text{fluorescence dsDNA}}}{{{\text{fluorescence dsDNA + }}\left( {{\text{fluorescence ssDNA*0}}{.4}} \right)}}$$

## Results and discussion

### Method development

**Fig. 1:**
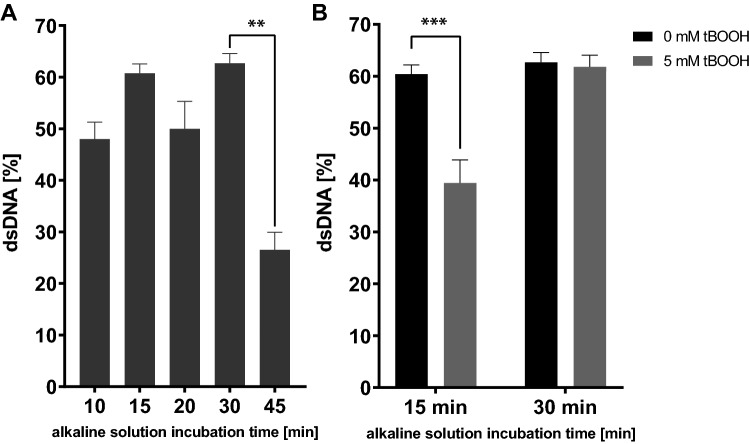
**A** Comparison of percentage of dsDNA to total DNA concentration of *C. elegans* with different incubation times of alkaline solution in control worms. Highest basal levels of dsDNA can be reached when incubating the alkaline solution for 15 min or 30 min. **B** Percentage of dsDNA to total DNA concentration when exposing worms to 5 mM tBOOH for 1 h. Significant differences between negative (0 mM tBOOH) and positive (5 mM tBOOH) controls can only be detected when using the alkaline solution for 15 min. Data are expressed as means ± SEM of at least 6 independent experiments. For statistical analysis, the unpaired *t* test was performed. **p < 0.01, ***p < 0.001

**Fig. 2:**
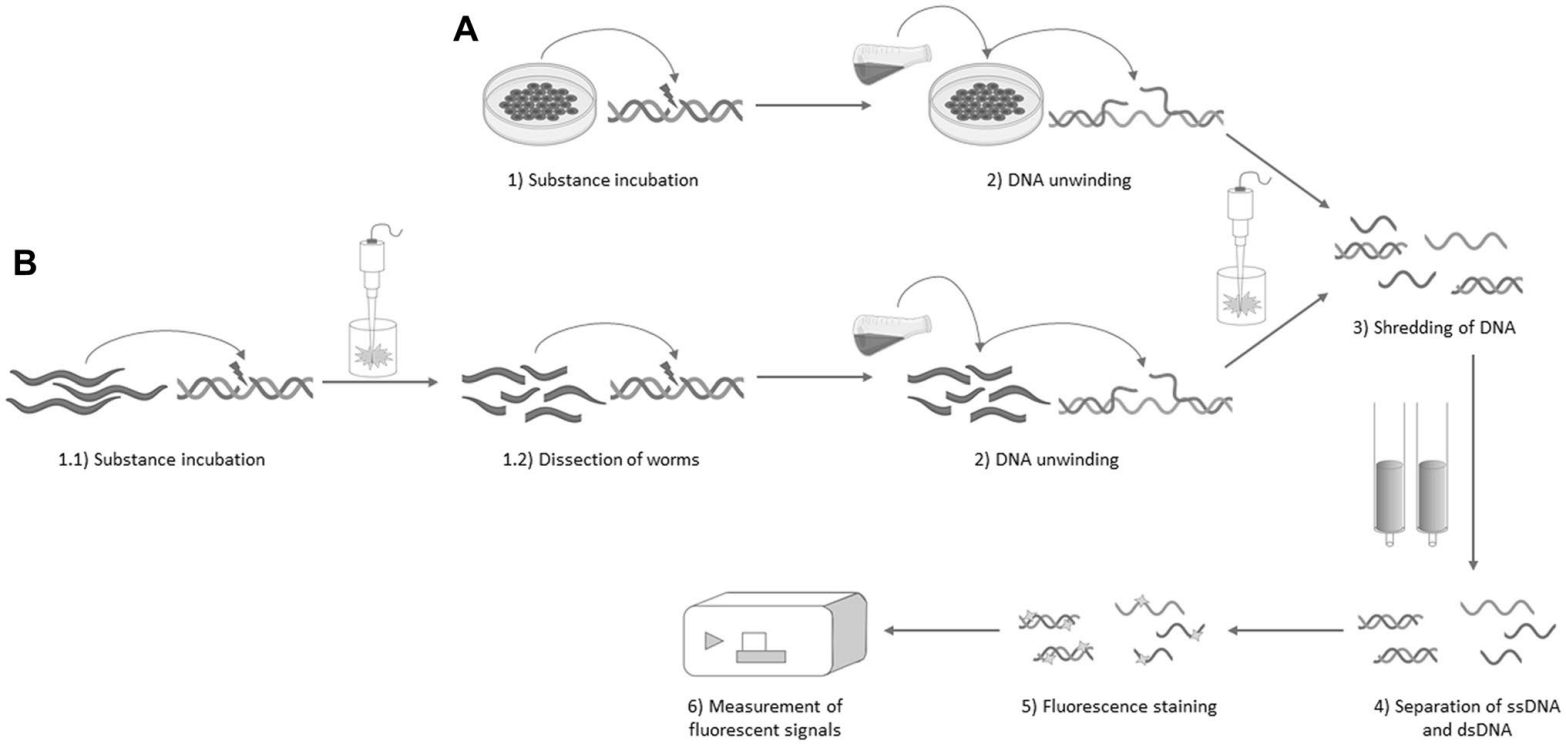
Schematic overview of the experimental setup for alkaline unwinding in **A** cell culture systems (Hartwig et. al (1993)) and **B** *C. elegans* samples. While the alkaline solution can be directly added to exposed cell samples, worms need to be made accessible using an ultrasonic disruptor before adding the alkaline solution. After DNA unwinding, both samples are then sonicated to shred the DNA. Using hydroxyapatite, dsDNA and ssDNA can be separated and finally quantified using fluorescence staining

Alkaline unwinding was initially developed for cell culture systems (Daniel et al. 1985; Hartwig et al. 1993) and is a frequently utilized genotoxicity test, even though it does not belong to the primal OECD genotoxicity tests. Nevertheless, alkaline unwinding is a well-established and rapid assay, which can be categorized as an indicator genotoxicity test (OECD 2015). Similar to the alkaline COMET assay (which is (in vitro) also not part of the primal OECD genotoxicity tests), using alkaline unwinding one can quantify DNA strand breaks caused by genotoxins via direct interactions with the DNA; alkali labile sites; or as a consequence of transient strand discontinuities resulting from nucleotide and base excision repair (Garberg et al. 1988; OECD 2016). In principle, the percentage of dsDNA is detected, which serves as a marker for genomic stability. A highly alkaline solution is used to enable the unwinding of the DNA and form ssDNA at sites of single-strand breaks. The quantification of dsDNA and ssDNA allows to draw conclusions regarding the amount of initial DNA single-strand breaks. Compared to the COMET assay, the procedure for alkaline unwinding does not call for isolation of intact cells, which can be complex in *C. elegans*. Despite being published (Imanikia et al. 2016), the COMET assay is experimentally demanding, as various cell types are present in worm extracts, background noise/matrix is high, few comparable data are present and results are challenging to reproduce. For alkaline unwinding, the DNA within the worms needs to be accessible to the alkaline solution, but it is not necessary to isolate intact cells. Lysing the worms is the critical step during this method since it is of crucial importance that DNA damages are not caused by the lysing process itself. We found that gentle sonification (Ultrasonic Processor, UP 100H, 2 × 20 s, in 1 mL liquid) works better for that purpose than using chemical dissipation of the cuticle (e. g. protein kinase K, pronase E, papain, Triton X-100), breaking of the cuticle with high pressure (French pressure cell press, as used for yeast cell lysis (Moore et al. 2016)) or slicing worms using syringes as described for germ cell isolation (Vagasi et al. 2017). Except for the gentle sonification, the other methods resulted in additional DNA damage as we detected only very low amounts of dsDNA (data not shown). Making use of transgenic worm strains did not facilitate the workflow in this particular case. The tested *bus-5* deletion mutant (DC19, bus-5(br19) X), which presents higher porosity, and therefore higher cuticle permeability for various substances compared to the wild type (N2) strain (Xiong et al. 2017), showed high levels of damage even in non-exposed controls. Mutant strains were therefore discarded as an alternative method for the lysing process and did not facilitate the accessibility of the alkaline solution. Experiments regarding the optimal incubation time with the alkaline solution can be seen in Fig. 1. The unwinding time was reduced from 30 min (used in cell samples) to 15 min as data indicate increased sensitivity of the worm DNA compared to DNA retrieved from cell culture (HepG2, BeWo b30) to the alkaline solution. This finding corresponds to earlier observations in a worm study pointing out that the *C. elegans* genomic DNA was detected to be degraded under alkaline electrophoresis condition used in a classical comet assay (Park et al. 2016). All following steps are identical to the in vitro setup (see Fig. 2), which was adapted from Hartwig et al. (1993). To keep DNA damage caused by the experimental setup to a minimum, worm samples were prepared in the dark and on ice after the compound treatment process was finished. For calculation of the percentage of dsDNA, the affinity of the Hoechst 33258 dye to dsDNA and ssDNA was tested. In contrast to cell samples, where a factor of 2.1 is present, in dsDNA of worms, the binding affinity is reduced by a factor of 0.4 of dsDNA compared to ssDNA. This, and the higher sensitivity of *C. elegans* DNA to the alkaline solution may indicate a slightly different coiling and stability of the DNA, which can also be a reason for the difficulties in the COMET assay, that has also been described by Park et al. (2016) as they found increased DNA damage in worms incubated with alkaline solutions at pH 12.3 for 30 min compared to controls. Additionally, both effects collectively may explain the high fluorescence following 30 min unwinding time (Fig. 1A). Nevertheless, when adapting the alkaline unwinding to worm DNA, this genotoxicity test proves to be meaningful, reliable, and practicable for high throughput.

### Positive controls

**Fig. 3:**
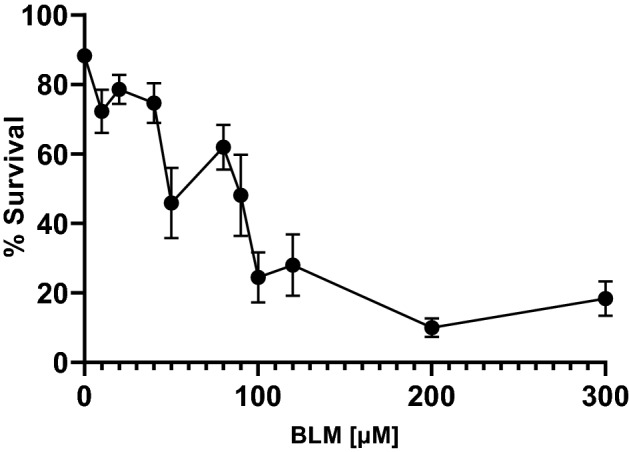
Percentage of survival after treatment with BLM for 1 h in a concentration range of 0–300 µM BLM. Survival of worms was quantified 24 h after exposure. Data are expressed as means ± SEM of at least 3 independent experiments

**Fig. 4:**
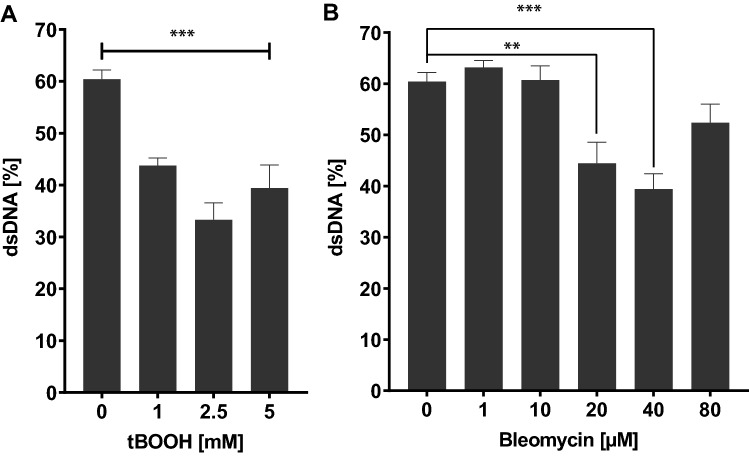
Percentage of dsDNA to total DNA concentration of *C. elegans* treated with tBOOH (**A**) or BLM (**B**) for 1 h. Data are expressed as means ± SEM of at least 3 independent experiments. For statistical analysis, the unpaired *t *test was performed. **p < 0.01, ***p < 0.001

For assay evaluation and verification, the positive controls tBOOH and BLM were used, as those chemicals are known to induce DNA strand breaks by different modes of action. TBOOH is a recognized inducer of oxidative stress and DNA damage in various in vitro and in vivo models, which is also effective and often applied in *C. elegans* (Helmer et al. 2021; Mersch-Sundermann et al. 2004; Xie et al. 2013). It is proposed that the oxidant causes the iron-dependent formation of *tert*-butoxyl (tBO·) and *tert-*butyl peroxyl (tBOO·) radicals, resulting in cellular redox imbalance associated with lipid peroxidation, Ca^2+^-dependent DNA cleavage, and apoptosis (Barton et al. 1998; Kruszewski et al. 2008; Martín et al. 2001). BLM is applied as an anti-cancer antibiotic. In comparison to tBOOH, BLM directly affects the DNA by causing athymic sites leading to single-strand splitting (Müller and Zahn 1976). Worms were incubated with both substances at sub-toxic concentrations for 1 h, which were determined in earlier studies (Neumann et al. 2020) or survival assays (Fig. 3). As expected, a highly significant and dose-dependent reduction of dsDNA is caused by exposing worms to tBOOH. Incubation of the substance of concentrations higher than the LD_25_ (2.5 mM tBOOH for 1 h (Neumann et al. 2020)), does not show any stronger effects, as the results show a maximum decrease of ~ 50% at this point (see Fig. 4A). Exposure of *C. elegans* to BLM does also lead to a dose-dependent significant induction of strand breaks and resulting decrease of dsDNA at 20 µM and 40 µM BLM (≤ LD_25_) compared to non-incubated control samples (Fig. 4B).

### Advantages of the model organism and alkaline unwinding compared to other genotoxicity tests

Factors that contribute to the genotoxicity potential of a compound on an organism are complex and often intertwined. Polymorphisms, age, gender, metabolic-enzyme expression, and lifestyle are a few examples that contribute to the vulnerability of an organism (Hsieh et al. 2019). While most of these attributes can be modeled in rodent experiments, they are not preferentially used for first screenings and indicator tests due to high time and cost intensity. Bacteria assays and cell culture systems are often used for initial investigations as they are less expensive and ethically acceptable for broad-spectrum screening. Prokaryotes and eukaryotes have developed complex biochemical responses to DNA damage that activate numerous processes like activation of repair mechanisms, transient cell cycle arrests, transcriptional upregulation of response proteins, and in metazoans apoptosis as the last resort. Considering that the DNA damage response and DNA damage checkpoints in higher eukaryotes are more complicated than those found in prokaryotes or unicellular eukaryotes, an easily accessible metazoan model organism is required to study the more complex aspects of genotoxicity (Stergiou and Hengartner 2004). The use of *C. elegans* is a valuable addition to genotoxicity assessment, as the nematode can be used as model organism for follow-up studies of in vitro testing and thus can improve the predictability for a possible genotoxic potential of a substance or treatment. With better predictability of genotoxicity, it would be possible to avoid unnecessary in vivo follow-up testing (and therefore replacement and reduction of animal testing). Genotoxicity testing in *C. elegans* is slowly getting more recognized, but validated methods are scarce. Besides the comet assay and rad-51 immunohistochemistry staining, very little is published for direct measurements of DNA damage in this model, and therefore a great need for additional, valid methods persists (Imanikia et al. 2016; Park et al. 2016; Toraason et al. 2021). Detecting the compound-induced genomic instability by the alkaline unwinding method in worms can be useful for (a) preliminary screening (high throughput), (b) as follow-up test of an in vitro positive result, (c) for mechanistic studies (the xenobiotic metabolism is highly conserved in *C. elegans*) and (d) exposure marker demonstrating that a substance is affecting the genomic stability. We are fully aware that the worm is a model organism with limitations, but it might provide a valuable addition to already existing strategies. The nematode is a multicellular and metabolizing organism, which is not given in cell lines or bacteria cultures (as used for the Ames test), where a metabolic activation system in form of liver-derived enzymes has to be additionally added via S9 fraction (OECD 2020). Additionally, the worm has a rapid life cycle and short generation time which will allow high throughput testing (Corsi et al. 2015; Nigon and Félix 2017). Most of the pathways involved in genomic integrity that are known through studies of bacteria, yeast, mammals, and human cell lines are also highly conserved in *C. elegans*, which makes the worm an experimental model greatly suited for research on processes involved in genomic stability (Elsakrmy et al. 2020; Gupta et al. 2021).

### Limitations, opportunities, and future directions

For method development, L4 larvae were chosen since they bear a fully developed DNA repair system but are not yet reproducing. Additionally, incubation of the positive controls was conducted in the absence of *E. coli* to avoid bacterial interferences. The two positive controls tBOOH and BLM were used as proof of principle for the AU assay in *C. elegans*. Both substances are validated DNA damage inducers (directly and indirectly) and no bioactivation is required for either of the substances. However, many other xenobiotics show only a genotoxic potential after being metabolized. Common examples are benzo[a]pyrene (BaP) and nitrosamines (Anttila et al. 2011; Arlt et al. 2012; Bodhicharla et al. 2014). The great advantage of the nematode compared to in vitro models is that the toxicokinetic pathways (phase I + II metabolism) needed for bioactivation are relatively similar to higher eukaryotes (Hunt 2017). Phase I metabolizing enzymes are necessary for the oxidation, reduction, and hydrolysis of xenobiotics and are broadly expressed in somatic cells of *C. elegans*. Over 85 cytochrome P450 isoforms have been identified in the nematode (compared to ~ 60 in humans) and many have been associated with xenobiotic metabolism (Menzel et al. 2001). Investigating substances that might only show their genotoxic potential after bioactivation is therefore very well possible in the nematode, but one must be aware of the differences that do exist between worms and mammals. For example, cytochrome P450 requires a heme cofactor and the coenzyme cytochrome P450 reductase. While *emb-8* is the worm´s homolog to the human P450 reductase, worms are not able to synthesize heme and need to scavenge this component from their diet (Hartman et al. 2021). Another metabolic difference can be found in the bioactivation of BaP, which causes the production of ROS and DNA adducts. In rodents and humans, BaP is predominantly metabolized by CYP1A1 leading ultimately to BaP-7,8-dihydrodiol-9,10-epoxide. While CYP1 enzymes do not exist in nematodes, studies still show the existence of DNA strand breaks in worms after BaP exposure, indicating a genotoxic potential of BaP caused by an alternative pathway of bioactivation (Abbass et al. 2021; Imanikia et al. 2016). An additional challenge of utilizing *C. elegans* for investigations regarding genomic stability is the current lack of data regarding enzyme activity, not only from metabolizing enzymes but also enzymes that are involved in DNA repair—making research in this area even more important.

The optimized and with acute genotoxins validated method provides a rapid genotoxicity evaluation in the model *C. elegans.* In future studies, the scope of the application will be extended to the investigations of known substances which are genotoxic upon metabolic activation, as well as being applied to more chronic exposure scenarios.
